# WIN 55,212-2 Modulates Antiviral, Inflammatory, and ER Stress Responses in Mayaro Virus-Infected Macrophages: Insights from RNA-Seq and In Vitro Studies

**DOI:** 10.3390/v18060662

**Published:** 2026-06-12

**Authors:** Lady Johana Hernández-Sarmiento, Juan Felipe Valdés-López, Silvio Urcuqui-Inchima

**Affiliations:** Grupo Inmunovirología, Facultad de Medicina, Universidad de Antioquia UdeA, Calle 70 No. 52-21, Medellín 050010, Colombia; ladyj.hernandez@udea.edu.co (L.J.H.-S.); felipe.valdes@udea.edu.co (J.F.V.-L.)

**Keywords:** Mayaro virus, macrophages, cannabinoids, inflammatory response, antiviral activity, stress, RNA-seq

## Abstract

Mayaro virus (MAYV) is an emerging arbovirus from the *Togaviridae* family where inflammation plays a central role in disease development. As the cause of Mayaro fever, MAYV triggers strong production of pro-inflammatory cytokines, which can result in long-lasting arthralgia in affected individuals. Macrophages are both targets for viral infection and key regulators of inflammatory responses. Human monocyte-derived macrophages (MDMs) are susceptible to MAYV infection in vitro and support productive viral replication. With no approved antivirals or vaccines, finding host-directed therapies is an urgent priority. Cannabinoids are compounds with antiviral and immunomodulatory properties, suggesting potential against MAYV infection. Here, we examined the effects of cannabidiol (CBD) and the synthetic cannabinoid WIN 55,212-2 on MAYV-infected MDMs in pre- and post-treatment conditions. Cells and supernatants were collected at 6 and 24 h post-infection (h.p.i). To understand the mechanisms involved, transcriptomic and functional analyses were performed at 24 h.p.i in the post-treatment setting, focusing on inflammatory, antiviral, and endoplasmic reticulum (ER) stress pathways. WIN 55,212-2 post-treatment significantly decreased viral replication at 24 h.p.i without any direct virucidal activity and was independent of type I interferon activation or interferon-stimulated gene induction, instead being linked to the modulation of ER stress signaling. Specifically, WIN 55,212-2 increased IRE-1α RNase activity, promoting the alternative splicing of sXBP1, while the integrated stress response appeared central to its antiviral effect. Additionally, WIN 55,212-2 downregulated inflammation-related genes and altered cytokine and chemokine production, counteracting the strong inflammatory response caused by MAYV. Remarkably, it also exerted broader immunomodulatory effects independent of infection.

## 1. Introduction

Mayaro virus (MAYV) is a zoonotic, arthropod-borne virus belonging to the genus *Alphavirus* within the family *Togaviridae*. Its genome, a single-stranded positive-sense RNA (ssRNA+), contains two open reading frames: the first encodes four non- structural proteins (nsP1–nsP4), involved in viral replication, while the second encodes the structural proteins necessary for virion assembly, including capsid (C), envelope glycoproteins (E1 and E2), and the cleavage products E3 and 6K [1]. First identified in 1954 in the serum of forest workers in Trinidad [2,3], MAYV is still classified as a neglected tropical disease. Nevertheless, the number of human infections has steadily increased over the past few decades across various regions worldwide, with a particularly notable rise in the Americas [4,5].

MAYV is primarily transmitted by mosquitoes of the *Haemagogus* genus [6]. However, other mosquito genera, including *Mansonia* [7], *Sabethes* [8], *Psorophora* [9], *Culex* [10,11], and *Aedes* [9,11,12,13], have also been identified as competent vectors, raising concerns about the potential for urban transmission. MAYV causes Mayaro fever (MAYF), a self-limited illness characterized by symptoms in about 90% of infected individuals. The incubation period is approximately 8 days [14], and like other arboviruses such as Chikungunya, Zika, and Dengue, infection with MAYV can cause symptoms including skin rash, fever, myalgia, diarrhea, retroorbital pain, dizziness, headache, and arthralgia [15,16,17]. Notably, the arthralgia caused by MAYV infection can last for months or even years, with 54% of patients experiencing chronic joint pain that mainly affects major joints [18]. MAYF can also lead to severe complications, including myocarditis, hemorrhagic fever, and neurological issues [19]. During the acute phase of MAYV infection, MCP-1 serum levels were significantly elevated compared to those of healthy controls, whereas IL-2 and IL-9 serum levels increased during the convalescent phase. In patients who developed persistent arthralgia, a sustained inflammatory profile was observed, characterized by high levels of IL-6, IL-7, IL-13, IL-17, VEGF, PDGF-BB, IL-1Ra, TNF-α, IFN-γ, and G-CSF. In contrast, IL-10 levels were markedly reduced throughout infection [20].

The sustained increase in cytokines and chemokines during MAYF indicated strong immune activation mainly driven by the innate immune system. Molecular data suggest that this response begins with the activation of pattern recognition receptors (PRRs), such as retinoic acid-inducible gene I (RIG-I), Toll-like receptors (TLRs), and inflammasomes, which detect pathogen-associated molecular patterns (PAMPs) [21]. The interaction between PRRs and PAMPs triggers a signaling cascade that boosts the expression of immune-responsive genes, especially those involved in inflammation, by activating nuclear factor-κB (NF-κB) [22]. Furthermore, the phosphorylation of various transcription factors, notably interferon regulatory factors (IRFs), leads to the secretion of interferons (IFNs), which activate antiviral responses by inducing interferon-stimulated genes (ISGs) [23,24]. This immune response is further enhanced by activation of the endoplasmic reticulum (ER) stress pathway, as shown by the upregulation of ER stress-related genes. This process activates the unfolded protein response (UPR), mediated by three main sensors: inositol-requiring enzyme 1 alpha (IRE1α), PKR-like ER kinase (PERK), and activating transcription factor 6 (ATF6) [25]. Collectively, these sensors initiate the cellular response to ER stress [26], a process closely linked to inflammation, particularly through NF-κB activation [27]. Additionally, ATF6 and the spliced form of X-box binding protein (sXBP1) promote the transcription of genes involved in the ER-associated degradation (ERAD) pathway [28].

Despite the potential threat posed by MAYV in tropical and subtropical regions worldwide, no prophylactic measures or specific antiviral therapies are currently available to prevent or treat MAYV infections [29]. Clinical management usually focuses on symptomatic relief, with analgesics and antipyretics, along with supportive care tailored to the patient’s clinical presentation. The urgent need for effective antiviral agents against MAYV has increased interest in plant-derived compounds that can disrupt the viral replication cycle. Cannabinoids, such as cannabidiol (CBD) and WIN 55,212-2, an aminoalkylindole synthetic derivative, have shown promise as therapeutic candidates because they can both reduce inflammation and inhibit viral replication. CBD has demonstrated antiviral activity against SARS-CoV-2 by inducing ER stress and stimulating IFN responses [30], as well as against the hepatitis C virus (HCV) [31]. However, CBD may have a dual effect on HIV-1, enhancing viral replication during early exposure while decreasing viral spread over more extended treatment periods [32]. Beyond its antiviral effects, CBD acts as an immunomodulator by reducing the production of IL-1β [33] and CCL-2, by inhibiting NF-κB activation [34].

Similarly, the synthetic cannabinoid WIN 55,212-2 has been shown to exert potent immunomodulatory effects. It inhibits the secretion of TNF-α, IL-1β, and IL-6, as well as CCL-2, CCL-5, and CXCL-10 [35]. Regarding antiviral activity, WIN 55,212-2 has been reported to inhibit HIV-1 entry by negatively regulating CCR5 [36] and to enhance the UPR, thus supporting cellular protein homeostasis [37]. Although these cannabinoids have been studied in various viral and inflammatory settings, the mechanisms by which CBD and WIN 55,212-2 modulate both immune and antiviral responses in monocyte-derived macrophages (MDMs) during MAYV infection remain largely unknown. We previously reported that MDMs are susceptible to in vitro infection with a Brazilian clinical isolate of MAYV, which triggers a strong pro-inflammatory and antiviral response [38], suggesting these mechanisms may be broadly relevant across alphavirus infections. In light of these findings, this study aimed to investigate the antiviral and immunomodulatory effects of WIN 55,212-2 in MAYV-infected MDMs through a combination of RNA sequencing (RNA-seq) analysis and in vitro assays. This approach enabled the comprehensive characterization of inflammatory, antiviral, and ER stress responses, validated by ELISA and/or RT-qPCR. Our findings highlighted the crucial role of WIN 55,212-2 signaling in determining the outcome of MAYV infection.

## 2. Materials and Methods

### 2.1. Ethics Statement

The Bioethics Research Committee at the “Institute of Medical Research of the Faculty of Medicine, University of Antioquia” approved the protocols for individual enrollment and sample collection on 26 April 2022 (CBI_008; CODE F-017-00). As previously described [38], these ethical approvals are in accordance with the principles outlined in the Declaration of Helsinki (1975, revised in 2013). All patients in this study provided written informed consent before blood collection. The study included RNA-seq samples from three healthy donors and in vitro experiments with four additional donors.

### 2.2. Cell Lines, MAYV Stock Production, and Viral Titration

A Brazilian clinical isolate of MAYV, generously provided by Professor Mauricio Nogueira (Faculdade de Medicina de São José do Rio Preto, São José do Rio Preto, São Paulo, Brazil), was propagated in Vero cells (ATCC CCL-81). Cells were maintained in Dulbecco’s Modified Eagle Medium (DMEM; Sigma-Aldrich, St. Louis, MO, USA) as previously described [38], and then infected with MAYV at a multiplicity of infection (MOI) of 0.1. Cultures were incubated at 37 °C with 5% CO_2_. Vital titers were determined by plaque assay in Vero cells.

### 2.3. Culture of Monocyte-Derived Macrophages

Human peripheral blood mononuclear cells (PBMCs) were isolated from leukocyte-enriched blood units obtained from healthy donors through the blood bank at the School of Microbiology, University of Antioquia (UdeA), Medellín, Colombia, using density gradient centrifugation with Lymphoprep (STEMCELL Technologies Inc., Vancouver, BC, Canada). Monocyte-derived macrophages (MDMs) were generated as previously described [38]. Briefly, CD14^+^ monocytes (5 × 10^5^ cells/well) were seeded in 24-well plates and incubated for 2 h in RPMI-1640 medium (Sigma-Aldrich) supplemented with 0.5% autologous serum, 4 mM L-glutamine, and 0.3% Na_2_CO_3_ at 37 °C with 5% CO_2_. Non-adherent cells were removed by washing twice with 1× PBS, and adherent monocytes were cultured in RPMI-1640 complete medium (10% FBS, 4 mM L-glutamine, 0.3% Na_2_CO_3_, and 1% antibiotic-antimycotic solution 100×) for 6 days under the same conditions. Each PBMC donor sample was processed independently.

### 2.4. Cannabinoids and ER Stress Inhibitors

Cannabidiol (CBD; Agilent; Catalog No. 5191-3924), (R)-(+)-WIN 55,212-2-mesylate (Sigma-Aldrich, St. Louis, MO, USA), ISRIB (SML0843, 500 nM), which blocks the integrated stress response (ISR) [39] and 4μ8C (SML0949, 25 µM), an inhibitor of IRE1α-mediated XBP1 splicing [40], were purchased from Sigma-Aldrich (St. Louis, MO, USA).

### 2.5. MAYV Infection of MDMs and Cannabinoid Treatments

**Figure 1:**
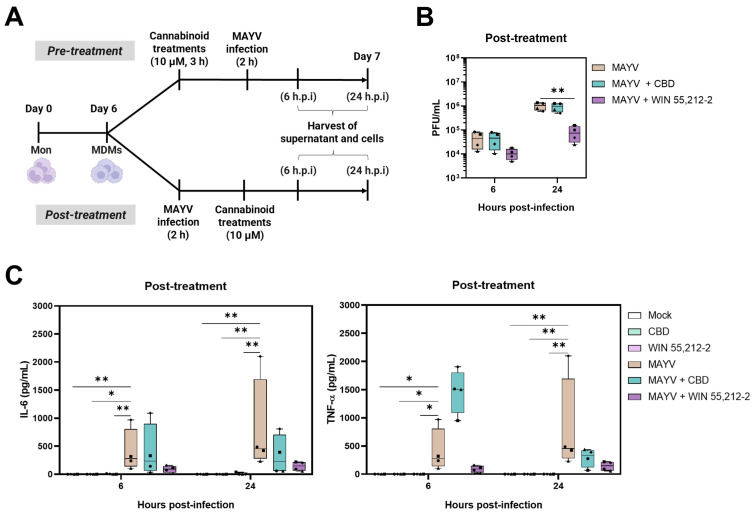
Effects of cannabinoid post-treatment on viral titers and production of IL-6 and TNF-α in MAYV-infected MDMs. Experimental design for cannabinoid treatments in MDMs (**A**). MDMs were subjected to two treatment approaches: pre-treatment and post-treatment. For post-treatment, MDMs were initially infected with MAYV (MOI 0.5) for 2 h or left uninfected, then treated with 10 µM cannabinoids (CBD or WIN 55,212-2) or left untreated at 37 °C with 5% CO_2_. Cells and supernatants were collected at 6 and 24 h.p.i and stored at −80 °C (**A**). Cell culture supernatants were collected, and viral replication was quantified by plaque assay (**B**). IL-6 and TNF-α production in MAYV-infected or uninfected MDMs, post-treated with WIN 55,212-2 or CBD, or left untreated, was quantified by ELISA (**C**). One-way ANOVA and Tukey’s tests were performed for normally distributed data at each time point, while Friedman and Dunn’s tests, depending on data distribution. (*n* = 4). Significance: *p* < 0.05 (*), *p* < 0.01 (**).

MDMs underwent two treatment conditions: pre-infection and post-infection (Figure 1A). For pre-infection treatment, cells were exposed to 10 μM CBD or WIN 55,212-2 for 3 h before infection with MAYV at an MOI of 0.5 in serum-free RPMI-1640. After 2 h of infection, cells were washed with 1× PBS to remove unbound virus, and fresh complete medium was added. For post-infection treatment, MDMs were first infected with MAYV at MOI 0.5 for 2 h, washed twice with 1× PBS, and then treated with CBD or WIN 55,212-2, followed by incubation at 37 °C with 5% CO_2_. In both conditions, cells and supernatants were collected at 6 and 24 h.p.i and stored at −80 °C. Untreated and uninfected MDMs served as the control group.

### 2.6. RNA-Seq and Transcriptomic Analysis

**Figure 2:**
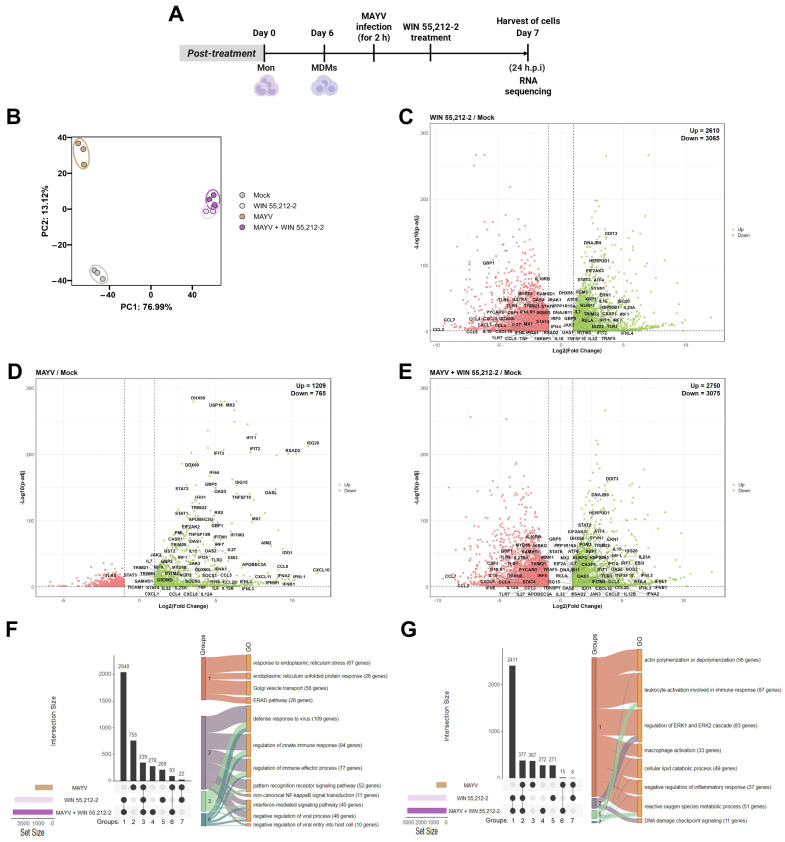
Transcriptional response of primary human MDMs to MAYV infection and WIN 55,212-2 post-treatment. MDMs (*n* = 3) were infected with MAYV (MOI 0.5) for 2 h, washed, and subsequently post-treated with WIN 55,212-2. Cells were collected at 24 h.p.i for RNA-sequencing analysis (**A**). Principal Component Analysis (PCA) plot shows distinct clustering of MAYV-infected MDMs (MAYV) and Mock samples, while WIN-treated MDMs (WIN 55,212-2) clustered together with MDMs treated with WIN 55,212-2 post-MAYV infection (MAYV + WIN 55,212-2), each with *n* = 3 biological replicates (**B**). Volcano plots of differentially expressed genes (DEGs). Volcano plots display DEGs in MDMs treated with WIN 55,212-2 (**C**), MAYV-infected MDMs (**D**), and MDMs treated with WIN 55,212-2 post-MAYV infection (**E**). The fold change (Log2) represents the difference in gene expression levels between the condition and Mock groups. Significance (−Log10) corresponds to [–Log10 (adjusted *p*-values)], indicating the statistical significance of each gene. A Log_2_FC of ≥1 or ≤−1 was considered indicative of upregulation or downregulation, respectively. UpSet plot showing the number of upregulated (**F**) and downregulated (**G**) DEGs unique to or shared among MAYV-infected MDMs, WIN-treated MDMs, and MDMs treated with WIN following MAYV infection. Gene Ontology enrichment analysis was performed to identify biological processes associated with each intersection.

Once the antiviral effect of WIN against the Mayaro virus was confirmed, we further investigated inflammation, antiviral activity, and ER stress responses in more detail. For this, MDMs (*n* = 3) were infected with MAYV (MOI 0.5) for 2 h, then washed, and subsequently, post-treated with 10 µM WIN 55,212-2. Cells were harvested at 24 h.p.i, and after plaque assay-confirmed viral replication, total RNA was extracted using the Direct-zolTM RNA Miniprep Plus (Zymo Research, Irvine, CA, USA) according to the manufacturer’s instructions. RNA samples were treated with DNase I (Zymo Research, Irvine, CA, USA), and RNA concentrations were measured with a Nanodrop spectrophotometer (Thermo Scientific, Waltham, MA, USA). For each sample, 300 ng of RNA was used for bulk RNA sequencing (RNA-seq; Figure 2A). Library preparation and sequencing were performed at Innomics Inc. (Sunnyvale, CA, USA) using the DNBSEQ platform. After sequencing, the sequencing facility converted the image data into raw reads and organized them into FASTQ files for each sample. To ensure dataset quality, FastQC analyses were performed as previously described [41]. Low-quality adapter sequences, poly-N stretches, and reads shorter than 70 bp were removed to obtain high-quality data. The remaining reads were aligned either to the human reference genome (GRCh38) using HISAT2 [42] or to the complete MAYV genome (NCBI Reference Sequence: NC_003417.1), enabling accurate and efficient mapping. Transcript assembly was conducted with StringTie [43], and feature quantification was carried out using the featureCounts function from the Subread package v3.0.0 [44], generating raw count values for each sample.

Differentially expressed genes (DEGs) were identified with the DESeq2 package in R (v4.2.0), applying two criteria: false discovery rate (FDR) < 0.05 and absolute Log2 fold Change (|Log_2_FC|) > 1. Principal Component Analysis (PCA) was performed using the plotPCA function from the DESeq2 package in R (v4.2.0) [45]. Volcano plots and Gene Ontology (GO) enrichment results were visualized with the ggplot2 library, and heatmaps were generated with the pheatmap package (v1.0.13). To investigate gene expression within signaling pathways, DEGs were mapped to pathways related to inflammation (including PRRs), antiviral response (IFNs and ISGs), and ER stress. These pathways were visualized in Cytoscape (v3.10.3) and integrated into a model representing the cellular responses assessed in this study. Spliced (sXBP1) and unspliced (uXBP1) isoforms were quantified from RNA-seq data across all experimental groups (Mock, WIN 55,212-2, MAYV, and MAYV + WIN 55,212-2), and the ratio of isoforms to total XBP1 reads (sXBP1 + uXBP1)/tXBP1 was calculated. Isoform distribution was visualized with stacked and proportional bar plots in R (v4.2.0) using the ggplot2 (v3.5.2), ggprism (v1.0.6), and cowplot (v1.1.3) packages. The raw count data and normalized RNA-seq TPMs are fully available in Supplementary Table S1.

### 2.7. Quantification of Cytokines and Chemokines

TNF-α, IL-1β, IL-6, IL-10, as well as CCL-2, CCL-5, and CXCL-8/IL-8 were measured in culture supernatants using the ELISA MAX™ Deluxe Set (BD Biosciences, San Jose, CA, USA), following the manufacturer’s instructions. The assay detection limit ranged from 0.5 to 10 pg/mL.

### 2.8. RNA Extraction, cDNA Synthesis, and RT-qPCR

Total RNA was extracted using TRIzol reagent (Invitrogen, Life Technologies, Carlsbad, CA, USA) following the manufacturer’s instructions. RNA concentration was measured with a NanoDrop-1000 spectrophotometer (Thermo Scientific, Wilmington, DE, USA). As previously described [38], complementary DNA (cDNA) synthesis was performed using the iScript^TM^ cDNA Synthesis Kit (Bio-Rad, Hercules, CA, USA). The mRNA expression levels of IFNβ1, IFNλ1, IL27p28, EBI3, STAT1, STAT3, SOCS1, APOBEC3A, ISG15, ISG20, Viperin, RIG-I, TLR7, NLRP3, CASP1, NFkB1, IkBα, IRE1α, uXBP1, sXBP1, BiP, ATF4, and GAPDH (housekeeping control) were quantified by RT-qPCR using SsoAdvanced™ Universal SYBR^®^ Green Supermix (Bio-Rad, Hercules, CA, USA) and gene-specific primers (Supplementary Table S2). Gene expression changes were calculated using the ΔΔCT method, expressed as Log_2_ Fold Change (Log_2_FC), and normalized to both GAPDH and the uninfected control. Cycle Threshold (Ct) values were determined through regression analysis within the linear amplification phase using Bio-Rad CFX Manager software v2.3 (5.3.022.1030). A Log_2_FC ≥ 1 or ≤−1 was considered up-or down-regulation, respectively.

### 2.9. Virucidal Assay

To evaluate the direct effect of WIN 55,212-2 on the viral particle, a virucidal assay was performed. Vero E6 cells were seeded overnight in 48-well plates at a density of 6 × 10^4^ cells/well and incubated at 37 °C with 5% CO_2_. MAYV inoculum (10^−1^) was prepared either alone or combined with WIN 55,212-2 (10 µM) and incubated for 45 min at 37 °C. Serial ten-fold dilutions were then made in DMEM. Cells were infected with 0.1 mL of each virus-compound mixture and incubated for 90 min at 37 °C with 5% CO_2_. After adsorption, the inoculum was removed and replaced with 0.5 mL of plaque assay medium (2% FBS, 3% [*v*/*v*] sodium carboxymethyl cellulose, and 2X DMEM (Sigma-Aldrich)) without WIN 55,212-2. Plates were further incubated for 3 days under the same conditions before plaque development and viral titration.

### 2.10. Treatment with ER Stress Inhibitors

For ER stress inhibitor assays, MDMs were pretreated with ISRIB (500 nM) or 4μ8C (25 μM) for 2 h. After pre-treatment, the inhibitors were removed, and the cells were washed before being infected with MAYV (MOI 0.5) for 2 h, as previously described [38]. Following infection, the unbound virus was removed by washing, and cultures were incubated with or without 10 µM WIN 55,212-2 in the continued presence of the respective inhibitor. Cultures were maintained at 37 °C with 5% CO_2_, and supernatants were collected at 24 h.p.i and stored at −80 °C for subsequent viral titration by plaque assay.

### 2.11. Statistical Analysis

Statistical analyses were conducted using R version 4.2.0 within RStudio (version 2024.04.2 Build 764) [45]. Normality was checked using the Shapiro–Wilk test, and homogeneity of variance was assessed with Levene’s test. The specific statistical tests used are described in the figure legends. Data are shown as box-and-whisker plots, which display the full range (from minimum to maximum) along with all individual data points. Statistical significance was defined as follows: *p* < 0.05 (*), *p* < 0.01 (**), and *p* < 0.001 (***).

## 3. Results

### 3.1. The Post-Treatment with WIN 55,212-2 Decreased MAYV Replication in MDMs

Based on our previous cytotoxicity assessment in MDMs using LIVE/DEAD flow cytometry, 10 μM of both CBD and WIN 55,212-2 were selected as non-cytotoxic concentrations for the MAYV infection assays [46]. Furthermore, since we previously established that MDMs are susceptible to MAYV infection in vitro [38], an MOI of 0.5 was selected for this study. Because this MOI supports productive viral replication while limiting the cytotoxic effects observed at higher MOIs, we examined whether treatment with CBD or WIN 55,212-2, administered either before or after infection (Figure 1A), could modulate MAYV replication. Pre-treatment with either compound did not protect MDMs from viral replication, and WIN 55,212-2 did not affect IL-6 or TNF-α production under these conditions, both of which were triggered by MAYV infection at 6 and 24 h.p.i (Figure S1). In contrast, post-treatment with WIN 55,212-2 significantly reduced viral titers at 24 h.p.i, but not at 6 h.p.i, while CBD did not affect replication (Figure 1B). Moreover, unlike CBD, post-treatment with WIN 55,212-2 slightly reduced IL-6 and TNF-α production at both 6 and 24 h.p.i, both of which were strongly induced by MAYV infection (Figure 1C).

### 3.2. Enrichment Analysis of Differentially Expressed Genes in Human MDMs During MAYV Infection and WIN 55,212-2 Post-Treatment

To investigate the molecular effects of WIN 55,212-2 post-treatment in MAYV-infected MDMs, bulk RNA-seq was performed at 24 h.p.i (Figure 2A), focusing on genes related to inflammatory, antiviral, and ER stress responses. PCA revealed a clear separation between MAYV-infected MDMs and Mock controls along PC2, which accounted for 13.12% of the variance (Figure 2B). Conversely, WIN-treated MDMs (WIN 55,212-2), whether alone or combined with MAYV infection (MAYV + WIN 55,212-2), clustered together and were separated from both Mock and MAYV-infected groups along PC1, explaining 76.99% of the variance. Together, PC1 and PC2 accounted for 90.11% of the total variance. These findings reveal distinct transcriptional signatures across the experimental groups, indicating that WIN post-treatment significantly influences the transcriptional landscape of MDMs.

As expected, viral infection and WIN 55,212-2 treatment induced significant transcriptional changes, as shown by the volcano plot analysis, which revealed distinct expression profiles across conditions. WIN 55,212-2 alone upregulated 2610 genes and downregulated 3065 genes (Figure 2C). MAYV infection led to 1209 genes being upregulated and 765 downregulated (Figure 2D). The combined treatment (MAYV + WIN 55,212-2) resulted in 2750 upregulated and 3075 downregulated genes (Figure 2E). These results indicate that WIN 55,212-2 has a more extensive and dominant effect on the transcriptional landscape of MDMs than MAYV infection alone. While MAYV infection mainly increased genes related to innate immunity, antiviral defense, and turned off stress-related genes in the ER, WIN 55,212-2, either alone or combined with MAYV, suppressed many innate immune and antiviral genes while activating those linked to ER stress signaling.

GO enrichment analysis was performed to identify biological processes associated with DEGs across the MAYV, WIN 55,212-2, and MAYV + WIN 55,212-2 conditions, separately analyzing upregulated and downregulated genes. An UpSet plot summarizes the distribution of upregulated DEGs unique to or shared among the three conditions (Figure 2F). The most significant intersection (Group 1: 2040 genes) included DEGs shared between WIN 55,212-2 treatment and MAYV + WIN 55,212-2 conditions, suggesting a highly conserved transcriptional program in MDMs primarily driven by WIN 55,212-2. These genes were significantly enriched in stress-related pathways, including the UPR, Golgi vesicle transport, and ERAD. In contrast, Group 2 (755 genes), which includes DEGs uniquely upregulated by MAYV infection, was enriched in antiviral processes such as defense response to viruses, regulation of innate immune responses, PRR signaling pathway, and IFN-mediated signaling. Notably, the pathway negative regulation of viral entry into host cells was exclusive to Group 2. These findings suggest that MAYV infection triggers a specific antiviral transcriptional response that persists, at least in part, even when WIN 55,212-2 is present. Interestingly, many of these processes overlapped with Group 3 (339 genes), representing DEGs common to all three conditions. Consistently, Group 6 (93 genes), shared between MAYV and the MAYV + WIN 55,212-2 conditions, was enriched in antiviral-related GO terms, including defense response to viruses, regulation of innate immune and effector processes, PRR signaling pathways, non-canonical NF-κB signaling, IFN-mediated pathways, and negative regulation of viral processes. These results indicate that WIN 55,212-2 treatment does not entirely suppress the antiviral response of MDMs but rather modulates or maintains specific aspects of it.

Figure 2G displays an UpSet plot summarizing the downregulated DEGs, highlighting those unique to or shared among the three experimental conditions. The most significant intersection (Group 1: 2411 genes), comprising DEGs shared between WIN 55,212-2 treatment and MAYV + WIN 55,212-2 conditions, revealed a conserved pattern of transcriptional repression enriched in biological processes, including actin filament dynamics, leukocyte activation, regulation of ERK1/2 signaling, macrophage activation, lipid breakdown, inflammation suppression, and ROS metabolism. Group 2 (377 genes), downregulated across all conditions, is enriched in ERK1/2 signaling regulation and DNA damage checkpoint pathways, indicating a core transcriptional signature common to both viral infection and WIN 55,212-2 exposures. Group 5 (271 genes) includes genes specifically linked to WIN 55,212-2, which are also associated with leukocyte activation and ROS metabolism, highlighting the consistent immunomodulatory effects of WIN 55,212-2. Group 7 (6 genes) shared between the MAYV and WIN 55,212-2 conditions was enriched in processes such as actin filament remodeling, macrophage activation, lipid breakdown, and inflammation suppression. These findings match the pattern observed in upregulated DEGs, supporting the notion that WIN 55,212-2 post-treatment modulates, rather than completely blocks, specific host responses, especially those related to cytoskeletal organization and inflammation.

### 3.3. Effects of WIN 55,212-2 Post-Treatment on the Expression of Inflammatory-Associated Genes in MAYV-Infected MDMs

**Figure 3:**
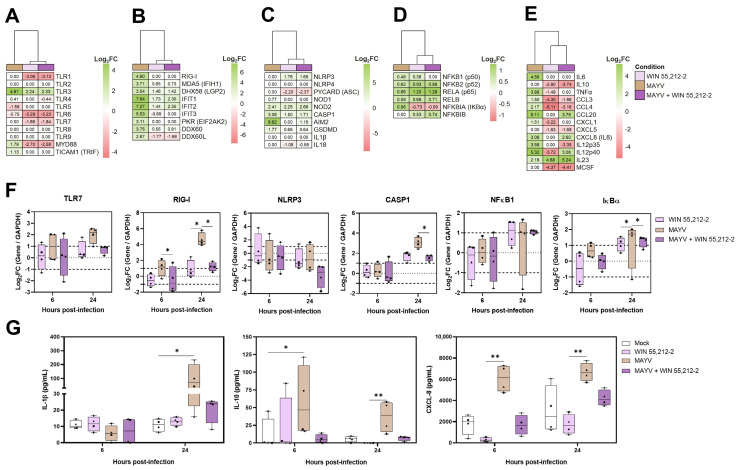
Modulation of WIN 55,212-2 post-treatment on the inflammatory response in human primary macrophages infected with MAYV. RNA-seq data from MDMs (*n* = 3) infected with MAYV and post-treated with WIN 55,212-2 at 24 h.p.i were analyzed. Heatmaps of gene expression in the inflammatory response. The heatmaps were generated in R (v4.2.0), where each column represents a condition: MDMs treated with WIN 55,212-2 (pink), MAYV-infected MDMs (beige), and MDMs treated with WIN 55,212-2 post-MAYV infection (purple). Each heatmap displays Log_2_FC values for selected genes, with upregulation (green) and downregulation (red) indicated. Genes with a *p*-value < 0.05 were included, and those with a Log_2_FC ≥ 1 or ≤−1 were considered significantly upregulated or downregulated, respectively. Heatmaps of Toll-like receptors (TLRs) and adapter proteins (**A**), RIG-like receptors (RLRs) and other cytosolic RNA sensors (**B**), NOD-like receptors (NLRs) and other cytosolic sensors (**C**), NF-κB transcription factors (**D**), and NF-κB target genes (**E**). Monocyte-derived macrophages were either infected with MAYV (MOI 0.5) for 2 h or left uninfected. Subsequently, they were post-treated with WIN 55,212-2 (10 µM) or left untreated at 37 °C with 5% CO_2_. Cells and supernatants were harvested at 6 and 24 h.p.i. Cell lysates were processed, and RT-qPCR was used to quantify the expression of selected genes. (**F**) Logarithm of fold change (Log_2_FC) ratios for TLR7, RIG-I, NLRP3, CASP1, NFkB1, and IkBα. A Log_2_FC of ≥1 or ≤−1 was considered indicative of upregulation or downregulation, respectively. (**G**) Cell culture supernatants were collected, and ELISA quantified the concentrations of cytokines and chemokines. Production of IL-1β, IL-10, and CXCL-8. Friedman and Dunn’s tests were performed. Data are presented as box-and-whisker plots showing the minimum and maximum values, with all individual data points displayed (*n* = 4). Significance: *p* < 0.05 (*), *p* < 0.01 (**).

Building on our previous enrichment analysis of DEGs in MDMs at 24 h.p.i in response to MAYV + WIN 55,212-2, we then focused specifically on genes involved in the inflammatory response. Genes with a *p*-value < 0.05 were selected for heatmap visualization, and those with a Log_2_FC ≥ 1 or ≤−1 were considered significantly up- or downregulated, respectively. Except for TLR3, which remained upregulated considerably across all conditions, TLR1, TLR6, and TLR7 were downregulated only in WIN 55,212-2-treated or infected-and-treated macrophages, but not in infected-only macrophages (Figure 3A). In contrast, TLR5 was downregulated solely in response to infection (Figure 3A). Furthermore, adaptor molecules of the TLR signaling pathway, including MYD88 and TICAM1 (TRIF), were upregulated in MAYV-infected MDMs, whilst in WIN 55,212-2-treated or MAYV + WIN 55,212-2 macrophages, they were either downregulated or remained unchanged.

RIG-I and other cytosolic RNA sensors, including MDA5, DHX58, IFITs, PKR, DDX60, and DDX60L, were strongly upregulated in MAYV-infected MDMs. In contrast, in WIN 55,212-2-treated or MAYV + WIN 55,212-2-treated MDMs, only DHX58, IFIT1, and IFIT2 remained significantly upregulated, although at lower levels than in infected cells, while the other sensors showed markedly reduced mRNA expression levels (Figure 3B). Regarding inflammasome-related transcription, NOD2, CASP1, AIM2, and GSDMD were significantly upregulated in MAYV-infected MDMs compared to both WIN 55,212-2 and MAYV + WIN 55,212-2 conditions, except for NOD2 (Figure 3C). Components of the NF-κB complex were slightly induced in all conditions. However, NFKBIA (IκBα), its inhibitory subunit, was markedly downregulated in both WIN 55,212-2-treated and in MAYV + WIN 55,212-2-treated MDMs (Figure 3D). Notably, MAYV infection triggered a strong proinflammatory transcriptional program, including cytokines and chemokines, which was downregulated in both infected-MDMs treated with WIN and in WIN-treated MDMs (Figure 3E). Additionally, WIN 55,212-2-treated or infected-and-treated MDMs also downregulated genes not induced by MAYV, such as IL10, CXCL5, and MCSF. Overall, these results suggest that WIN post-treatment not only counteracts infection-driven inflammatory signaling but also exerts additional immunoregulatory effects independent of viral infection.

Next, we validated the expression of selected DEGs identified in the transcriptomic analysis by RT-qPCR (TLR7, RIG-I, NLRP3, CASP1, NFκB1, and IκBα) and cytokine/chemokine production using ELISA (IL-1β, IL-10, and CXCL-8/IL-8) at 6 and 24 h.p.i. As shown in Figure 3F, TLR7 expression decreased slightly in WIN-treated MDMs (*p* < 0.1) at 24 h.p.i. RIG-I mRNA levels increased in MAYV-infected MDMs but significantly reduced in MAYV + WIN 55,212-2–treated cells at both 6 and 24 h.p.i, whereas CASP1 mRNA expression was significantly decreased only at 24 h.p.i compared with MAYV-infected cells. In contrast, NLRP3 and NF-κB1 expression remained unchanged at both time points, whilst IκBα mRNA levels were significantly downregulated in WIN 55,212-2-treated or infected-and-treated MDMs, regardless of infection status.

As expected, MAYV infection significantly increased IL-1β production at 24 h.p.i, while IL-10 and CXCL-8/IL-8 levels were markedly elevated at both 6 and 24 h.p.i (Figure 3G). Conversely, IL-1β levels slightly decreased at 24 h.p.i following WIN 55,212-2 treatment, whereas IL-10 production showed a downward trend in level at both time points in infected and post-treated MDMs. Similarly, CXCL8/IL-8 levels tended to decrease at 6 h.p.i in infected and post-treated cells, and were significantly reduced in WIN 55,212-2-treated MDMs at both 6 and 24 h.p.i (Figure 3G). Overall, these findings suggest that WIN 55,212-2 post-treatment modulates the inflammatory response in MAYV-infected MDMs by altering the expression of innate immune receptors and cytokine and chemokine production, thereby affecting the host response to infection.

### 3.4. Effects of WIN 55,212-2 Post-Treatment on the Antiviral Response in MAYV-Infected MDMs

**Figure 4:**
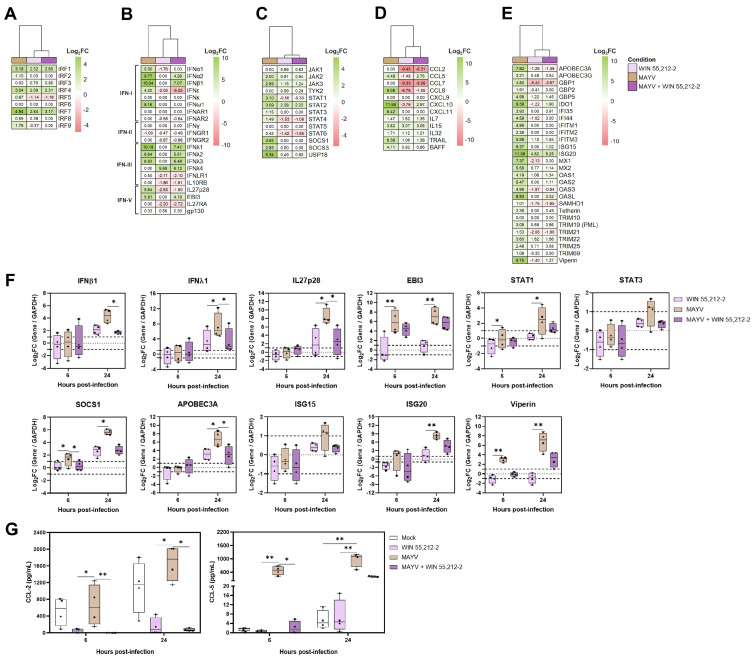
Effects of WIN 55,212-2 post-treatment on the antiviral response in human primary macrophages infected with MAYV. RNA-seq data from MDMs (*n* = 3) infected with MAYV and post-treated with WIN 55,212-2 at 24 h.p.i were analyzed. Heatmaps of gene expression involved in the antiviral response. The heatmaps were generated in R (v4.2.0), where each column represents a condition with a color: MDMs treated with WIN 55,212-2 (pink), MAYV-infected MDMs (beige), and MDMs treated with WIN 55,212-2 post-MAYV infection (purple). Each heatmap displays Log_2_FC values for selected genes, with upregulation (green) and downregulation (red) indicated. Genes with a *p*-value < 0.05 were included, and those with a Log_2_FC ≥ 1 or ≤−1 were considered significantly upregulated or downregulated, respectively. Heatmaps of Interferon regulatory factors (IRFs) (**A**), Interferons (IFNs) and their receptors (**B**), JAK-STAT mediators and negative regulators (**C**), cytokines and chemokines dependent on STAT signaling (**D**), and Interferon-stimulated genes (ISGs) (**E**). MDMs were either infected with MAYV (MOI 0.5) for 2 h or left uninfected. Subsequently, they were post-treated with WIN 55,212-2 (10 µM) or left untreated at 37 °C with 5% CO_2_. Cells and supernatants were harvested at 6 and 24 h.p.i. Cell lysates were processed, and RT-qPCR was used to quantify the expression of selected genes. (**F**) Logarithm of fold change (Log_2_FC) ratios for IFNβ1, IFNλ1, IL27p28, EBI3, STAT1, STAT3, SOCS1, APOBEC3A, ISG15, ISG20, and Viperin. (**G**) Cell culture supernatants were collected, and ELISA quantified the concentrations of chemokines. Production of CCL-2 and CCL-5. Friedman and Dunn’s tests were performed. Data are presented as box-and-whisker plots showing the minimum and maximum values, with all individual data points displayed (*n* = 4). Significance: *p* < 0.05 (*), *p* < 0.01 (**).

Since post-treatment with WIN 55,212-2 significantly reduced MAYV replication in MDMs, we aimed to identify gene expression changes using RNA-seq to understand how WIN 55,212-2 influences the host cell response to the virus. The heatmap of all analyzed genes revealed two distinct clusters: one associated with MAYV infection and another linked to WIN 55,212-2 treatment, which included both WIN 55,212-2 alone and MAYV + WIN 55,212-2 (Figure 4A–E). This indicated that WIN 55,212-2 reprograms gene expression away from the pattern induced by MAYV. Specifically, MAYV infection significantly increased mRNA levels of IRF1, IRF2, IRF4, IRF7, and IRF9, while WIN 55,212-2 and MAYV + WIN 55,212-2 caused slight decreases in IRF1, IRF4, and IRF7. Instead of elevating IRF5, these treatments significantly downregulated its expression (Figure 4A). While MAYV infection strong induced the expression of type 1 IFNs (IFN-α1, IFN-α2, IFN-β1, IFN-ε, IFN-ω1), type III IFNs (IFN-λ1–3), and type V IFNs (IL27p28 and EBI3 subunits), their expression was maintained in the MAYV + WIN 55,212-2 condition, but at slightly lower levels, except for IFN-α1, IFN-ε, and IL27p28, which were downregulated (Figure 4B). Furthermore, type II IFN (IFN-γ) remained unchanged, except IFNGR1, which was significantly downregulated in MAYV-infected MDMs (Figure 4B). Both WIN 55,212-2 and MAYV + WIN 55,212-2 did not also induce but instead significantly downregulated the expression of IFNLR1, IL10RB, and IL27RA (Figure 4B). Additionally, WIN treatment significantly increased IFN-λ4 in both WIN 55,212-2 and MAYV + WIN 55,212-2 (Figure 4B). While MAYV infection strongly induced genes related to the JAK/STAT pathway, including JAK2, JAK3, STAT1, STAT2, STAT3, STAT4, SOCS1, SOCS3, and USP18, this expression was modestly decreased in both WIN-treated MDMs and MAYV + WIN 55,212-2 condition (Figure 4C). STAT4 and STAT6 expression were significantly downregulated in WIN-treated and MAYV + WIN 55,212-2, whilst mRNA levels of STAT3, SOCS1, and SOCS3 remained unchanged.

Our transcriptomic analysis also confirmed that MAYV infection significantly upregulated genes associated with JAK/STAT signaling pathway, including CCL5, CCL8, CXCL10, CXCL11, IL7, IL15, IL32, TRAIL, and BAFF (Figure 4D). Interestingly, in MDMs treated with WIN 55,212-2 or MAYV + WIN 55,212-2, mRNA levels of CCL2, CCL7, and CCL8 were significantly decreased. Meanwhile, expression of CCL5, CXCL10, TRAIL, and BAFF was reduced in MAYV + WIN 55,212-2-treated MDMs compared to those infected with MAYV alone (Figure 4D).

Next, we assessed the expression of selected ISGs encoding antiviral proteins induced during MAYV infection, as well as the modulatory effects of WIN post-treatment. MAYV infection triggered a robust antiviral response by significantly upregulating ISGs such as APOBEC3A, APOBEC3G, GBP1, GBP2, GBP5, IDO1, IFI35, IFI44, IFITM1, IFITM2, IFITM3, ISG15, ISG20, MX1, MX2, OAS1, OAS2, OAS3, OASL, SAMHD1, Tetherin, TRIM19, TRIM21, TRIM22, TRIM25, TRIM69, and Viperin (Figure 4E). Interestingly, most of these ISGs were not induced but suppressed in MDMs treated with WIN 55,212-2 or MAYV + WIN 55,212-2. Notably, GBP1 was the most strongly and significantly downregulated gene, while other ISGs, such as APOBEC3A, SAMHD1, and TRIM21, also decreased considerably, though to a lesser degree. Meanwhile, a subset of ISGs, including GBP5, IFITM3, ISG20, OAS1, and TRIM22, remained significantly upregulated, albeit at lower levels compared to MAYV infection.

The transcriptomic results were validated by measuring the expression of representative DEGs associated with the antiviral response using RT-qPCR and by assessing STAT1-dependent chemokines using ELISA at 6 and 24 h.p.i. As expected, viral infection increased mRNA levels of antiviral genes such as IFNβ1, IFNλ1, IL27p28, and EBI3, but these were downregulated at 24 h.p.i in both WIN 55,212-2-treated and infected-and-treated MDMs, except for EBI3 (Figure 4F). At 6 h.p.i, the expression of these genes remained stable across all groups, except for EBI3. Although STAT1 levels increased significantly at both time points, STAT3 remained unchanged following infection; STAT1 mRNA levels decreased significantly after WIN 55,212-2 treatment at 6 and 24 h.p.i. While SOCS1, APOBEC3A, and ISG20 were upregulated in MAYV-infected MDMs, SOCS1 expression notably decreased in WIN 55,212-2-treated or in MAYV + WIN 55,212-2 conditions, at 6 h.p.i, with a trend toward reduction at 24 h.p.i. APOBEC3A expression was significantly reduced in both WIN 55,212-2-treated and infected-and-treated MDMs at 24 h.p.i. ISG20 mRNA levels were markedly decreased in WIN-treated MDMs compared to those in infected and treated MDMs (Figure 4F). Conversely, whilst Viperin mRNA levels significantly increased after MAYV infection at 6 and 24 h.p.i, its levels were significantly decreased in WIN 55,212-2-treated MDMs but not in MAYV + WIN 55,212-2 MDMs. The ISG15 mRNA levels remained unchanged across the three conditions (Figure 4F). Overall, these findings suggest that the antiviral effects of WIN 55,212-2 after infection are not primarily driven by enhancing ISG expression or directly activating the IFN response, suggesting the involvement of an alternative regulatory pathway.

The production of CCL-2 and CCL-5 increased in MAYV-infected MDMs at both 6 and 24 h.p.i. In contrast, CCL-2 production significantly decreased in WIN 55,212-2-treated and infected-and-treated MDMs at both time points, whereas CCL-5 showed a significant reduction only at 6 h.p.i in WIN 55,212-2-treated cells (Figure 4G). These results suggest that WIN 55,212-2 exerts a selective immunomodulatory effect on MAYV-induced chemokine production.

### 3.5. WIN 55,212-2 Does Not Exert a Virucidal Effect on MAYV Replication In Vero E6 Cells

Since ISG induction was not associated with WIN treatment, we next investigated whether its effect might be due to a virucidal mechanism, which is defined as the direct inactivation of viral particles before or during entry into host cells. To test this, Vero E6 cells were infected with tenfold serial dilutions of MAYV, either alone or with 10 µM WIN 55,212-2. The absence of significant differences in viral replication between untreated and WIN-treated infected cells (Figure S2) showed that WIN 55,212-2 does not exert a virucidal effect against MAYV.

### 3.6. Effects of WIN 55,212-2 Post-Treatment on ER Stress Response in MAYV-Infected MDMs

**Figure 5:**
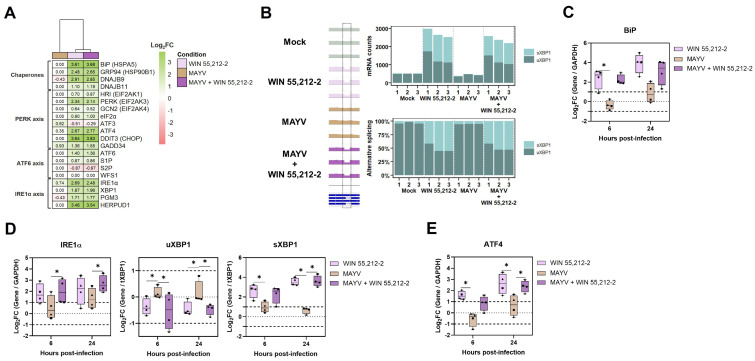
Regulation of the Stress response by WIN 55,212-2 post-treatment in human primary macrophages infected with MAYV. RNA-seq data from MDMs (*n* = 3) infected with MAYV and post-treated with WIN 55,212-2 at 24 h.p.i were analyzed. Heatmaps of gene expression involved in the stress response. The heatmaps were generated in R (v4.2.0), where each column represents a condition with a color: MDMs treated with WIN 55,212-2 (pink), MAYV-infected MDMs (beige), and MDMs treated with WIN 55,212-2 post-MAYV infection (purple). Heatmap displays Log_2_FC values for selected genes, indicating upregulation (green) or downregulation (red). Genes with a *p*-value < 0.05 were included, and those with a Log_2_FC ≥ 1 or ≤−1 were considered significantly upregulated or downregulated, respectively. (**A**) Heatmap of genes categorized according to their roles in the UPR, including chaperones and folding assistants (BiP, GRP94, DNAJB9, and DNAJB11); components of the PERK axis (HRI, PERK, GCN2, eIF2α, ATF4, DDIT3, and GADD34); the ATF6 axis (ATF6, S1P, S2P, and WFS1); and the IRE1α axis (IRE1α, XBP1, PGM3, and HERPUD1). (**B**) Analysis of XBP1 splicing by the IRE1α RNase. Reads corresponding to spliced and unspliced XBP1 were identified and quantified for Mock, WIN 55,212-2-treated MDMs (WIN 55,212-2), MAYV-infected MDMs (MAYV), and MDMs treated with WIN 55,212-2 post-MAYV infection (MAYV + WIN 55,212-2). The percentage of alternatively spliced reads in the RNA-seq samples was plotted, and the ratio of spliced (sXBP1) and unspliced (uXBP1) XBP1 reads to total XBP1 reads ((sXBP1 + uXBP1)/tXBP1) was calculated. MDMs were either infected with MAYV (MOI 0.5) for 2 h or left uninfected. Subsequently, they were post-treated with WIN 55,212-2 (10 µM) or left untreated at 37 °C with 5% CO_2_. Cells were harvested at 6 and 24 h.p.i, and RT-qPCR was used to quantify gene expression. Logarithm of fold change (Log_2_FC) ratios for BiP (**C**), IRE1α, uXBP1, sXBP1 (**D**), and ATF4 (**E**). Friedman and Dunn’s tests were performed. Data are presented as box-and-whisker plots showing the minimum and or maximum values, with all individual data points displayed (*n* = 4). Significance: *p* < 0.05 (*).

Although this study did not find any antiviral effect of CBD, recent reports suggest that CBD inhibits SARS-CoV-2 replication by inducing host ER stress [30]. Therefore, our transcriptomic analysis focused on ER stress, because the transcriptional profile shows that, unlike MAYV infection, which suppresses ER stress, both WIN 55,212-2- and MAYV + WIN 55,212-2-treated MDMs activate the ER stress response (Figure 5A). In both conditions, WIN 55,212-2 caused a strong upregulation of chaperones BiP (HSPA5), GRP94 (HSP90B1), and DNAJB9, indicating activation of the UPR. Within the ISR, mainly mediated by the PERK axis under ER stress, HRI (EIF2AK1), PERK (EIF2AK3), GCN2 (EIF2AK4), eIF2α, ATF4, DDIT3 (CHOP), and GADD34 were consistently upregulated in both WIN-treated groups, while ATF3 was downregulated.

Within the ATF6 axis, which regulates ERAD components, ATF6 itself and S1P were upregulated in both WIN 55,212-2- and MAYV + WIN 55,212-2-treated MDMs, while S2P was downregulated, and WFS1 remained unchanged (Figure 5A). In the same way, the IRE1α axis was strongly activated under both WIN 55,212-2 conditions, as indicated by significant upregulation of IRE1α, XBP1, PGM3, and HERPUD1. Collectively, these findings demonstrate that WIN 55,212-2 strongly activates ER stress signaling through the PERK and IRE1α branches of the UPR, while also increasing ATF6 transcript levels. When activated, IRE1α removes a specific 26-nucleotide intron from the uXbp1 mRNA, producing the spliced form (sXBP1) [47]. The sXBP1 transcript encodes an active transcription factor that enhances ERAD components and ER chaperones [48]. Then, we examined our RNA-seq data to determine the ratio of sXBP1 and uXBP1 mRNA relative to total XBP1 mRNA, calculated as (sXBP1 + uXBP1)/tXBP1 (total XBP1). As shown in Figure 5B, sXBP1 splicing was induced in both WIN-treated and MAYV + WIN-treated MDMs but was suppressed during MAYV infection alone. This pattern suggests that WIN treatment stimulates ERAD activation in MDMs.

To validate these transcriptomic results, we performed RT-qPCR at 6 and 24 h.p.i to assess mRNA levels of chaperones, the IRE1α axis, and ATF4. As shown in Figure 5C, BiP mRNA expression was consistently increased in both WIN 55,212-2- and MAYV + WIN 55,212-2-treated MDMs at 6 and 24 h.p.i, whereas it decreased in MAYV-infected MDMs at both time points (Figure 5D). Additionally, both WIN 55,212-2-treated and MAYV + WIN 55,212-2-treated MDMs exhibited increased IRE1α mRNA levels at both 6 and 24 h.p.i, reaching statistical significance in the MAYV + WIN 55,212-2 condition compared with MAYV-infected cells. In contrast to IRE1α, uXBP1 mRNA levels were markedly increased in MAYV-infected MDMs but were significantly reduced at both time points in WIN 55,212-2-treated and infected-and-post-treated MDMs (Figure 5D). Conversely, sXBP1 (Figure 5D) and ATF4 (Figure 5E) transcripts were significantly decreased in MAYV-infected MDMs but increased in WIN 55,212-2-treated and MAYV + WIN 55,212-2-treated MDMs at 24 h.p.i. Overall, the RNA-seq data and their validation indicate activation of the IRE1α-XBP1 branch of the UPR in response to WIN 55,212-2 treatment, suggesting that this pathway may contribute to enhanced host defense mechanisms.

### 3.7. Impact of ISR and IRE1α-XBP1 Inhibition on MAYV Infection

**Figure 6:**
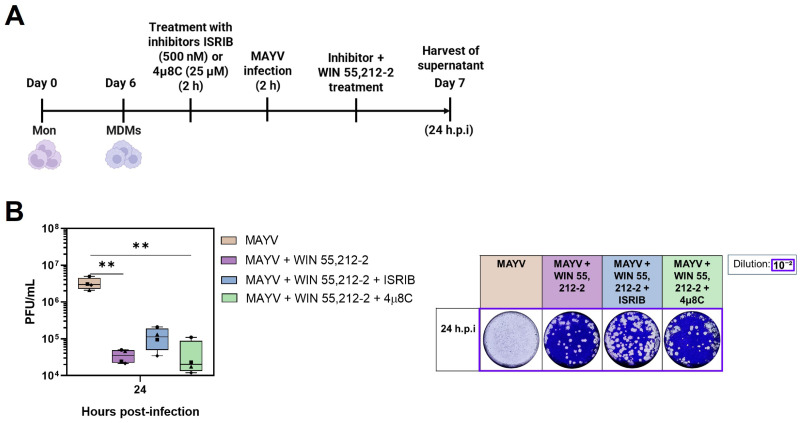
Effects of ISRIB and 4u8C in MAYV-infected MDMs following post-treatment with WIN 55,212-2. MDMs were either treated with integrated stress response inhibitors, ISRIB (500 nM) or 4u8C (25 µM), or left untreated, and incubated at 37 °C with 5% CO_2_ for 2 h. Following this, the cells were either infected with MAYV at MOI of 0.5 for 2 h or left uninfected. Subsequently, the MDMs were treated with WIN 55,212-2 (10 µM) or left untreated and maintained at 37 °C with 5% CO_2_. Supernatants were collected at 24 h.p.i (**A**), and a plaque assay was performed to quantify the viral replication (**B**). Representative plaque images from at least 4 independent experiments were obtained using crystal violet. Friedman and Dunn’s tests were performed. Data are presented as box-and-whisker plots showing the minimum and maximum values, with all individual data points displayed (*n* = 4). Significance: *p* < 0.01 (**).

Given the strong ER stress activation induced by WIN 55,212-2 treatment, we hypothesized that pharmacological modulation of this pathway could be a potential strategy for controlling MAYV replication. After confirming MDMs viability and the expression of sXBP1, ATF4, and DDIT3 mRNA following drug treatment, we assessed the inhibitory effects of ISRIB, which blocks the ISR [39], and 4μ8C, which prevents IRE1α-mediated XBP1 splicing [49], in MAYV-infected and MAYV + WIN 55,212-2-treated MDMs at 24 h.p.i (Figure 6A). As shown in Figure 6B, ISRIB treatment showed a trend toward significance, while 4μ8C had no impact on restoring MAYV replication compared to the MAYV + WIN 55,212-2 condition (Figure 6B). Overall, these results indicate that WIN 55,212-2 enhances the antiviral response to MAYV mainly through activation of the ISR branch of the UPR, with only minor contribution from IRE1α RNase activity.

### 3.8. WIN 55,212-2 Decreases MAYV ORF1 and ORF2 Transcript Levels in MDMs

**Figure 7:**
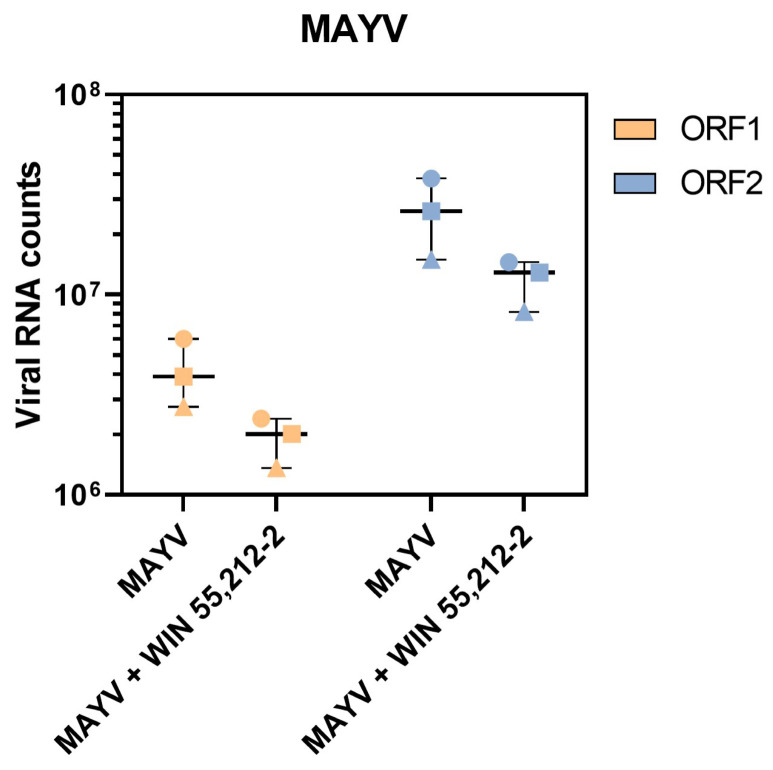
Viral mRNA counts of MAYV ORF1 and ORF2 in human MDMs. MDMs (*n* = 3) were infected with MAYV (MOI 0.5) for 2 h, washed, and subsequently post-treated with WIN 55,212-2 (10 µM). At 24 h.p.i, cells were harvested, and viral RNA levels were quantified by RNA sequencing for ORF1 (orange) and ORF2 (blue). Data are presented as box-and-whisker plots (min to max), with all individual values shown (*n* = 3). The horizontal line inside each box represents the median. Statistical analysis was performed using a paired Student’s *t*-test.

As shown in Figure 7, RNA-seq analysis revealed that MAYV-infected MDMs contained more than 10^7^ viral mRNA reads for ORF2, whereas those for ORF1 exceeded 10^6^. Treatment with WIN 55,212-2 (either alone or after viral infection) tended to reduce transcript levels for both MAYV ORFs compared to untreated infected MDMs. This reduction affected both ORF1 and ORF2 to a similar degree. In both conditions, ORF2 transcript levels consistently surpassed those of ORF1, reflecting the abundant production of subgenomic RNA encoding structural proteins. ORF1 encodes the nonstructural polyprotein necessary for viral replication, while ORF2 encodes the structural proteins essential for virion assembly and release [50]. The consistent ORF2-to-ORF1 ratio under WIN 55,212-2 treatment suggests a general reduction in viral RNA accumulation, with no clear evidence of selective effects on genomic versus subgenomic RNA expression.

## 4. Discussion

Despite recurrent MAYV outbreaks in the Americas [51,52,53,54], no specific treatment or vaccine is currently available to control the infection. As an arthritogenic virus [20], MAYV triggers a strong inflammatory response that drives macrophage overactivation and the release of mediators that contribute to tissue damage. This immune dysregulation is key to the arthritis and myositis observed in arthritogenic alphaviruses [55,56]. We previously demonstrated that macrophages are susceptible to MAYV infection, which triggers both inflammatory and antiviral responses [38]. Given macrophages’ dual roles in viral control and immunopathology, cannabinoids have emerged as promising immunomodulatory agents due to their ability to regulate inflammatory and antiviral pathways. In line with this, our previous work on CHIKV demonstrated that post-treatment with WIN 55,212-2 reduced inflammation, modulated ER stress-related genes, and exerted antiviral activity through mechanisms that are not yet fully understood [46]. In this study, we evaluated the immunomodulatory and antiviral effects of WIN 55,212-2 in macrophages infected with MAYV. Post-treatment with WIN 55,212-2 significantly decreased viral load by nearly one log at 24 h.p.i. At the same time, CBD showed no antiviral activity under similar conditions, in contrast to reports of CBD’s effects against SARS-CoV-2 [30] and HCV [31]. This discrepancy may be explained by the context-dependent nature of CBD’s antiviral activity, which varies according to the specific virus, cell type, treatment regimen, and host pathways engaged. Under our experimental conditions, the selected non-cytotoxic concentration of CBD might have been insufficient to induce the stress-adaptive or antiviral mechanisms required to restrict MAYV replication in MDMs. Conversely, WIN 55,212-2 triggered a broader UPR/ISR-associated transcriptional response, which likely accounts for its distinct antiviral efficacy.

To better understand how WIN 55,212-2 influences immune and antiviral responses, we conducted an in-depth analysis of inflammatory, antiviral, and ER stress responses by combining RNA-seq and in vitro assays in MAYV-infected MDMs.

Transcriptomic profiling revealed that WIN 55,212-2 alone triggered extensive transcriptional remodeling, affecting as many genes as in MAYV-infected and post-treated MDMs (2610 vs. 2750), whereas infection alone upregulated only 1209 genes. This highlights the broad regulatory influence of WIN on macrophage homeostasis and immunity, even in the absence of disease. Activation of cannabinoid receptors triggers pathways that modulate ER stress, PRR, and IFN signaling, as well as negatively regulating viral replication. This suggests that WIN 55,212-2 primes macrophages toward an antiviral, stress-adaptive state, potentially contributing to reducing viral replication and inflammation.

The DEG enrichment analysis showed that WIN post-treatment reprograms macrophage responses more profoundly than MAYV infection alone. Genes induced by WIN 55,212-2, with or without infection, were strongly associated with UPR, ER stress, and ERAD pathways, thereby maintaining an antiviral response while suppressing excessive NF-kB and JAK/STAT-driven inflammation. These adaptive pathways are known to restore proteostasis and mitigate virus-induced cell stress in other viral infections [49,57]. Conversely, MAYV-infection alone predominantly activated an inflammatory and ISG network. Consistent with this, heatmap clustering revealed that WIN-treated and infected/post-treated MDMs grouped separately from MAYV-infected-only MDMs, indicating a transcriptional shift away from inflammation toward a stress-adaptive phenotype.

In agreement with previous reports [21], genes upregulated only by MAYV infection were enriched for antiviral and innate immune functions involving PRR signaling (TLR3/TRIF, RIG-I/MDA5, IFIT1-3, DDX60) and the IFN response, as well as genes negatively regulating viral entry, indicating an intrinsic antiviral state. However, in MAYV-infected cells, WIN alters the expression of inflammation-associated genes, regulating cytokine and chemokine production and potentially shaping the macrophage-driven immune response. The downregulation of PRRs and NF-κB-related transcripts, a key driver of pro-inflammatory gene expression [22], may explain the slight reduction in inflammatory mediators, including CXCL-8/IL-8, CCL-2, CCL-5, IL-6, and TNF-α. These cytokines and chemokines contribute to the immunopathogenesis and chronic arthritis associated with alphavirus infection and rheumatoid arthritis [58,59]. TNF-α and IL-6 also function as pyrogenic cytokines [60], while CCL-2, CCL-5, and CXCL-8/IL-8 facilitate the recruitment of immune cells [61,62]. Furthermore, WIN post-treatment also reduced IL-10 levels, an anti-inflammatory cytokine essential for regulating host immune responses [63]. The reduced IL-10 expression is consistent with previous studies showing dose-dependent inhibition by WIN in LPS/IFN-γ-stimulated macrophages [64]. In addition to NF-κB signaling, several of the cytokines and chemokines modulated by WIN are classically regulated through JAK/STAT-dependent pathways [65,66]. NF-κB and JAK/STAT signaling pathways are known to operate in a tightly interconnected manner, where NF-κB-driven cytokine production [22,67], such as IL-6 subsequently activates STAT signaling to shape the magnitude, duration, and resolution of inflammatory responses [68,69]. Therefore, the coordinated downregulation of NF-κB-related transcripts together with reduced expression of STAT-dependent cytokines suggests that WIN may attenuate macrophage inflammatory responses through combined modulation of the NF-κB/JAK-STAT signaling axis. Studies using cannabinoid compounds distinct from WIN 55,212-2 support the broader relevance of JAK/STAT-NF-κB crosstalk in cannabinoid-mediated immune regulation. In this context, a review by Peyravian et al. summarizes multiple experimental studies demonstrating that cannabidiol (CBD) modulates immune responses through inhibition of key components of the JAK/STAT signaling pathway. Specifically, transcriptomic analyses in human mesenchymal stem cells, together with functional studies in immune cell models, revealed downregulation of STAT-dependent cytokines such as IL-6, IL-10, IFN-γ, and TNF-α following CBD treatment where these effects were accompanied by suppression of NF-κB-mediated transcription, underscoring a coordinated regulation of inflammatory cytokine networks through JAK/STAT and NF-κB pathways [70]. Altogether, WIN reprograms macrophages toward a cytoprotective, stress-adaptive state while limiting excessive proinflammatory signaling, potentially reducing immunopathology and oxidative stress-related tissue damage.

Among downregulated genes, those suppressed by WIN 55,212-2 (3065) or by infection combined with treatment (3075) were primarily associated with cytoskeletal remodeling, leukocyte activation, ERK1/2 signaling, and lipid catabolism processes often linked to proinflammatory and metabolic activation states [71]. These findings are consistent with previous evidence demonstrating that the cannabinoid receptor 2 agonist, JWH-133, downregulates the expression of proteins involved in NF-κB signaling and the Nrf2-mediated oxidative stress response in HIV-1-infected MDMs [72]. Furthermore, shared genes between infection and WIN treatment were enriched in ERK and DNA damage checkpoint signaling, suggesting a coordinated cellular adaptation integrating stress and immune regulation. Similarly, Pérez-Diego et al. (2025) reported that WIN 55,212-2 protects the airway epithelial barrier during rhinovirus A16 infection by modulating oxidative metabolism and STAT6 phosphorylation [73]. Together, these findings indicate that WIN 55,212-2 enhances cellular protection by activating the UPR while reducing inflammation and preserving antiviral capacity.

**Figure 8:**
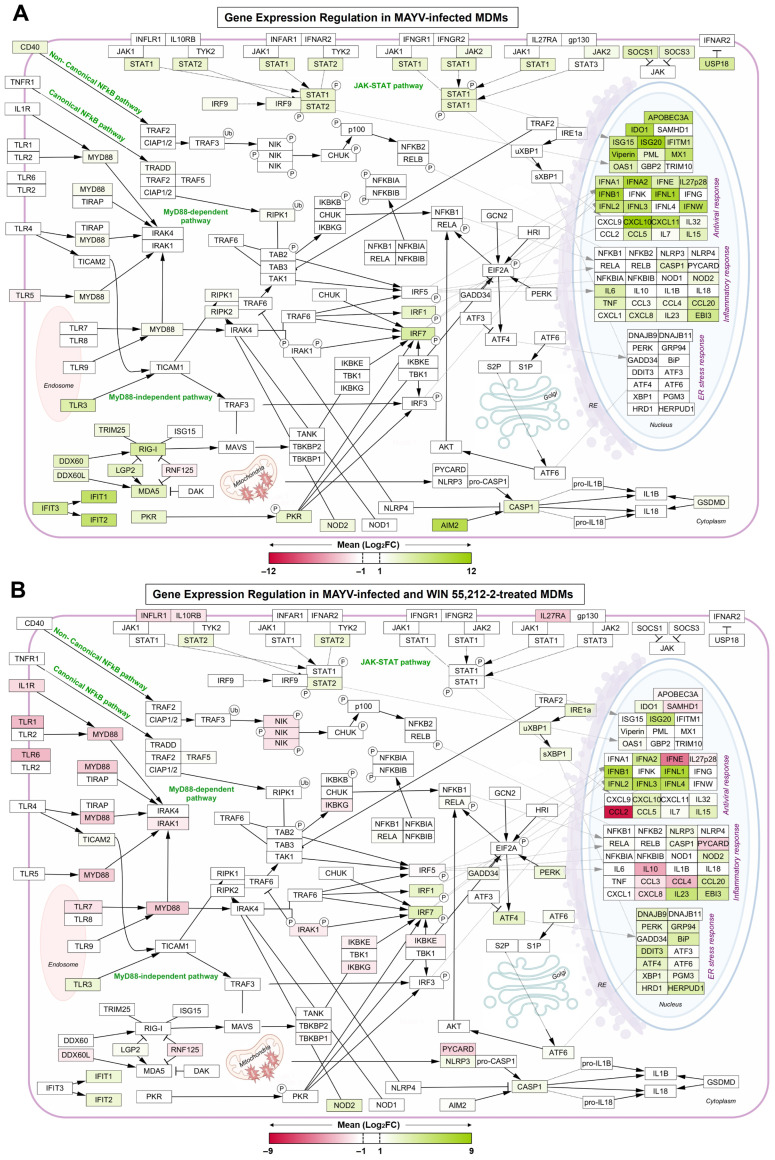
Working model of pathway integration of inflammatory, antiviral, and stress responses in MAYV-infected MDMs with or without WIN 55,212-2 post-treatment. The working model summarizes gene expression within signaling pathways associated with inflammatory, antiviral, and stress responses in MAYV-infected MDMs (**A**) and MDMs treated with WIN 55,212-2 post-MAYV infection (**B**). Genes with a *p*-value < 0.05 and a Log_2_FC ≥ 1 or ≤−1 were included. DEGs were uploaded to Cytoscape (v3.10.3) to visualize previously described pathways, which were then integrated into a model designed to reflect the responses evaluated in this study. Genes are represented as rectangles, with colors indicating upregulation (green) or downregulation (red) based on the mean Log_2_FC.

Network visualization in Cytoscape supported this dual effect. MAYV infection primarily upregulates genes related to classical antiviral and pro-inflammatory pathways through PRRs, NF-κB, and ISG cascades, without evidence of ER stress gene activation (Figure 8A). In contrast, WIN treatment, either alone or post-infection, markedly reprograms this transcriptional profile. As shown in Figure S3, strongly engaged PERK-ATF4-DDIT3 and IRE1α-XBP1 pathways, reflecting a shift from canonical IFN signaling toward an ISG. This indicates that WIN exerts a basal immunoregulatory and stress-adaptive effect on macrophages, independent of viral infection. When combined with MAYV infection (Figure 8B), WIN further changed the transcriptional response, significantly reducing the expression of all ISGs induced by viral infection or downregulating them, thereby suppressing PRR-MYD88-NF-κB signaling, while inducing stress response genes not engaged during infection alone. This finding aligns with Pérez-Diego et al. (2023), who discovered that WIN reprograms macrophages metabolically and epigenetically to suppress LPS-induced inflammation [64]. Thus, cannabinoid receptor agonists can rewire macrophage function to limit harmful inflammation and restrict viral replication through non-canonical mechanisms.

The antiviral activity of WIN appears to depend on ISR activation rather than ISG upregulation. WIN 55,212-2 induced multiple UPR branches, particularly the PERK-eIF2α-ATF4-DDIT3 axis, which likely contributes to translational control limiting viral replication. Inhibition of the ISR with ISRIB partially restored viral replication, confirming that ISR contributes to WIN’s antiviral action. While studies on MAYV and its modulation of ER stress are limited, comparisons with CHIKV, a closely related alphavirus, offer valuable insights. In the case of CHIKV, we also observed that WIN upregulated IRE1α and sXBP1 mRNA levels in both infected and uninfected MDMs [46], indicating its role in modulating cellular stress response pathways. Furthermore, it has been reported that CHIKV infection reduces IRE1α and sXBP1 expression, potentially as a viral strategy to suppress the UPR and favor viral replication, an effect consistent with the limited XBP1 mRNA splicing observed by Fros et al. (2015) during CHIKV infection [74]. In line with these observations, other studies have shown that alphaviruses and unrelated RNA viruses exploit ER stress pathways in a context-dependent manner. For example, CHIKV has been described as preferentially activating the ATF6 and IRE1α branches of the UPR while suppressing PERK signaling, thereby promoting nsP4 expression and replication, and upregulating GRP78, a regulator of ER homeostasis [75,76,77]. Similarly, activation of the IRE1α-XBP1 axis has been linked to enhanced replication of SARS-CoV-2, hepatitis B virus, and Zika virus [78,79,80,81]. These findings underscore that UPR signaling can exert either pro-viral or antiviral roles depending on the cellular and viral context, and suggest that pharmacological manipulation of ER stress pathways may alter the outcome of infection.

Conversely, inhibition of the IRE1α-XBP1 pathway with 4μ8C did not affect viral replication, suggesting that ISR, but not IRE1α splicing, is critical for the antiviral response. Notably, the IRE1α-XBP1 system has been reported to enhance viral replication [79,82]. This supports a model in which ISR activation reprograms translation and transcription to restrain viral replication, as was previously reported [83]. Since ISR reduces cap-dependent translation under stress [74], it may shift the cellular program towards homeostatic recovery rather than the classical IFN-driven antiviral state. WIN’s concurred reduction in both pro- and anti-inflammatory cytokines, including IL-10, underscores the complex modulation of this balance via stress signaling.

Interestingly, RNA-seq analysis also showed that WIN 55,212-2 post-treatment significantly decreased the levels of MAYV ORF1 and ORF2. As ORF1 encodes the nonstructural polyprotein required for RNA replication and ORF2 encodes structural proteins [49], their simultaneous reduction suggests that WIN broadly inhibits viral RNA synthesis or stability without altering the genomic/subgenomic RNA ratio. This suggests that WIN may limit the efficiency of the replication cycle by generally impairing RNA synthesis or stability. This supports an additional mechanism whereby WIN-induced ER stress activates ERAD and autophagy-like quality-control processes that degrade the viral replication complex. Moreover, activation of the IRE1α-XBP1 axis may trigger regulated IRE1-dependent decay of viral RNAs during prolonged ER stress. Given that alphavirus replication relies on ER-derived membranes, cannabinoid-mediated disruption of ER homeostasis or lipid biosynthesis could hinder the formation of replication factories. This dual mechanism, involving antiviral stress signaling and metabolic disruption, may represent a broader antiviral strategy of cannabinoids that deserves further investigation in other RNA viruses.

## 5. Conclusions

Our findings indicate that MAYV induces an inflammatory response in macrophages, which can be modulated by WIN 55,212-2. Additionally, WIN 55,212-2 shifts the macrophage transcriptional profile toward stress adaptation and reduces pro-inflammatory signaling, supporting the antiviral response in MAYV-infected human macrophages. This profile could be therapeutically beneficial for limiting immunopathology during MAYV infection, provided antiviral defenses, especially ER stress-mediated responses, are maintained, at least partially through the IRE1α-XBP1 and ISR pathways. However, detailed studies in animal models are necessary to evaluate the potential and efficacy of WIN 55,212-2 as an antiviral treatment.

## Data Availability

The original contributions presented in this study are included in the article/Supplementary Materials. Further inquiries can be directed to the corresponding author.

## Supplementary Materials

The following supporting information can be downloaded at: https://www.mdpi.com/article/10.3390/v18060662/s1, Figure S1: Effects of cannabinoid pre-treatment on Mayaro viral titers and IL-6 and TNF-α production in MAYV-infected MDMs. MDMs were pre-treated with 10 µM of cannabinoids (CBD or WIN 55,212-2) or left untreated, then incubated at 37 °C with 5% CO_2_ for 3 h. The cultures were infected with MAYV (MOI 0.5). Supernatants were collected, and viral replication was measured by plaque assay (**A**). IL-6 and TNF-α levels (**B**) in pre-treated and untreated MDMs, infected or uninfected, were quantified by ELISA. Friedman and Dunn’s tests were used for analysis. Data are shown as box-and-whisker plots with all individual data points (*n* = 4), displaying minimum and maximum values. Significance levels: *p* < 0.1 (.), *p* < 0.05 (*), *p* < 0.01 (**), *p* < 0.001 (***). A *p*-value < 0.1 indicates a trend toward significance (.); Figure S2: Assessment of the Virucidal Activity of WIN 55,212-2 against MAYV in Vero E6 cells. Vero E6 cells were infected with ten-fold serial dilutions of the MAYV and MAYV combined with WIN 55,212-2 (10 µM) at 37 °C with 5% CO_2_. Viral titers were measured by plaque assay, and representative images of the plaques were captured after crystal violet staining. Friedman and Dunn’s tests were performed. Data are presented as box-and-whisker plots showing the minimum and maximum values, with all individual data points displayed (*n* = 4). Significance: *p* < 0.1 (.), *p* < 0.05 (*), *p* < 0.01 (**), *p* < 0.001 (***). A *p*-value < 0.1 was considered marginally significant, indicating a trend toward significance (.); Figure S3: Working model of pathway integration of inflammatory, antiviral, and stress responses in MDMs post-treated with WIN 55,212-2. The working model summarizes gene expression within signaling pathways associated with inflammatory, antiviral, and stress responses in WIN 55,212-2-treated-MDMs. Genes with a *p*-value < 0.05 and a Log2FC ≥ 1 or ≤−1 were included. DEGs were up-loaded to Cytoscape (v3.10.3) to visualize previously described pathways, which were then integrated into a model designed to reflect the responses evaluated in this study. Genes are represented as rectangles, with colors indicating upregulation (green) or downregulation (red) based on the mean Log2FC. Table S1: Raw count data and normalized RNA-seq TPMs. Table S2: Gene-specific primer pairs used in this study.
