# Supplementary material for: WIN 55,212-2 Modulates Antiviral, Inflammatory, and ER Stress Responses in Mayaro Virus-Infected Macrophages: Insights from RNA-Seq and In Vitro Studies
Source: Viruses. 2026 Jun 12;18(6):662. doi: 10.3390/v18060662 (PMC13307677; doi:10.3390/v18060662)
Supplement: Supplementary file 1 [file viruses-18-00662-s001.zip › Table S2.pdf]

Supplementary Table S2. Gene-specific primer pairs used in this study

Gene Symbol	Forward Sequence (5'-3')	Reverse Sequence (5'-3')
GAPDH	TCGGAGTCAACGGATTTGGT	TGAAGGGGTCATTGATGGC
RIG-I	TAAGGGGATGATGGCAGGTG	TGGGCCAGTTTTCCTTGTCT
TLR7	TTACCTGGATGGAAACCAGCTACT	TCAAGGCTGAGAAGCTGTAAGCTA
NLRP3	GGACTGAAGCACCTGTTGTGCA	TCCTGAGTCTCCCAAGGCATTC
CASP1	GCTGAGGTTGACATCACAGGCA	TGCTGTCAGAGGTCTTGTGCTC
NFkB1	GCAGCACTACTTCTTGACCACC	TCTGCTCCTGAGCATTGACGTC
IkBα	TGTGATCACCAACCAGCCAG	AGTCTCGGAGCTCAGGATCA
IFN-β1	CGCCGCATTGACCATCTA	GACATTAGCCAGGAGGTTTCTCA
IFN-λ1	GGTGACTTTGGTGCTAGGCT	TGAGTGACTCTTCCAAGGCG
IL27p28	GAGCAGCTCCCTGATGTTTC	AGCTGCATCCTCTCCATGTT
EBI3	TGGCTCCCTACGTGCTCAAT	GAGGGTCGGGCTTGATGATGT
STAT1	GGCAAAGAGTGATCAGAAACAA	GTTCAGTGACATTCAGCAACTC
STAT3	GAGAAGGACATCAGCGGTAAG	AGTGGAGACACCAGGATATTG
SOCS1	CACTTCCGCACATTCCGTTC	CACGCTAAGGGCGAAAAAGC
APOBEC3A	CATCCGGGCCCAGGCATAA	TCTTGACCGAGGTGCCATTG
ISG15	GGTGGACAAATGCGACGAAC	TCGAAGGTCAGCCAGAACAG
ISG20	TGCTGTGCTGTACGACAAGTT	CTCTTTCAGTGCCTCTAGCC
Viperin	AAATGCGGCTTCTGTTTCCAC	TTGATCTTCTCCATACCAGCTTCC
IRE1α	CCAACATCTCCTGGCTCTTTCAC	GTCAGGATGCTGTGATAGGCGT
tXBP1	TGAAAAACAGAGTAGCAGCTCAGA	CCCAAGCGCTGTCTTAACTC
uXBP1	CAGACTACGTGCACCTCTGC	CTGGGTCCAAGTTGTCCAGAAT
sXBP1	GCTGAGTCCGCAGCAGGT	CTGGGTCCAAGTTGTCCAGAAT
BiP	CTGTCCAGGCTGGTGTGCTCT	CTTGGTAGGCACCACTGTGTTC
ATF4	TTCTCCAGCGACAAGGCTAAGG	CTCCAACATCCAATCTGTCCCG
DDIT3	GGTATGAGGACCTGCAAGAGGT	CTTGTGACCTCTGCTGGTTCTG
