# Supplementary figures and images for: WIN 55,212-2 Modulates Antiviral, Inflammatory, and ER Stress Responses in Mayaro Virus-Infected Macrophages: Insights from RNA-Seq and In Vitro Studies
Source: Viruses. 2026 Jun 12;18(6):662. doi: 10.3390/v18060662 (PMC13307677; doi:10.3390/v18060662)

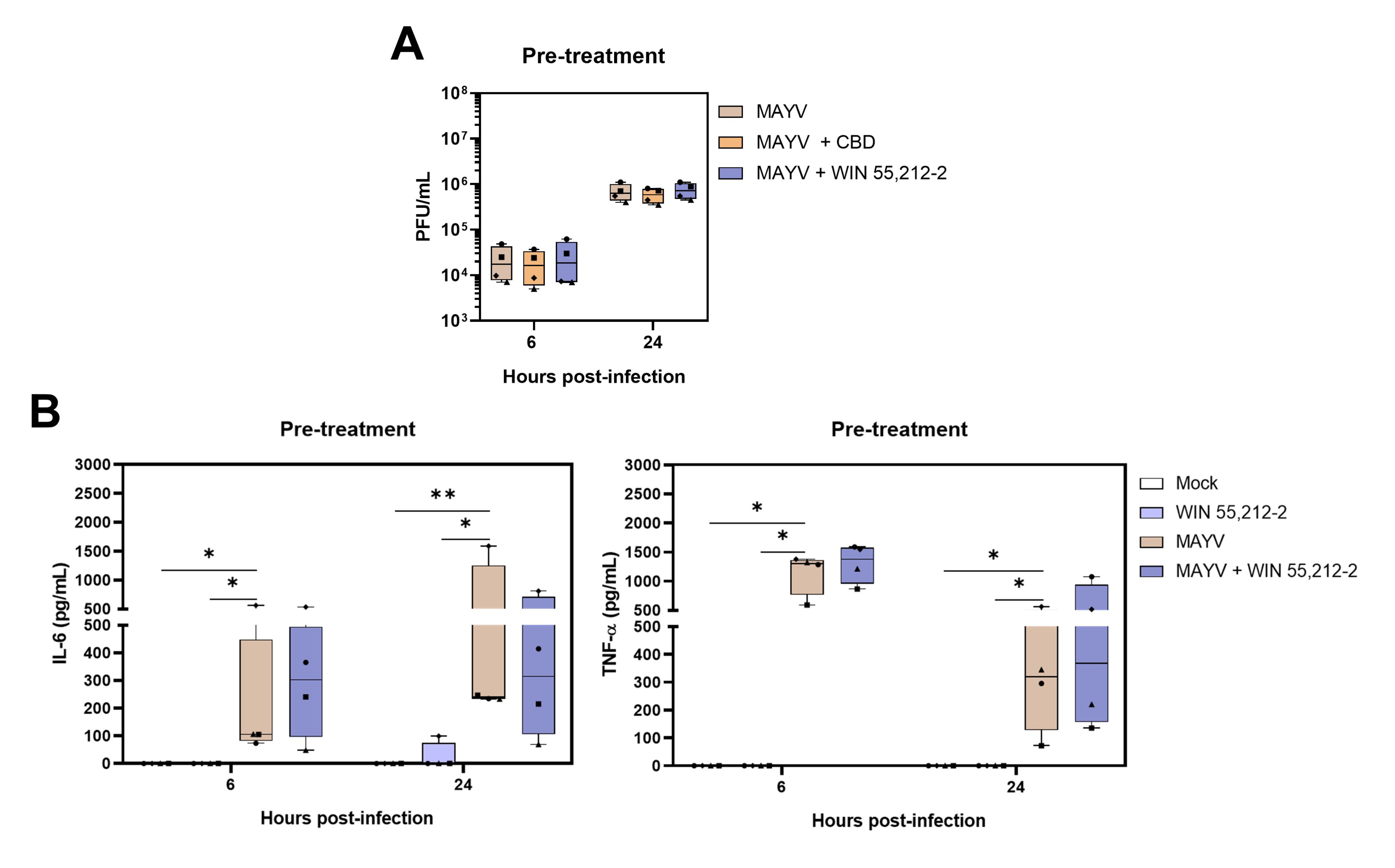

Supplement: Supplementary file 1 [file viruses-18-00662-s001.zip › Figure S1 .jpg]

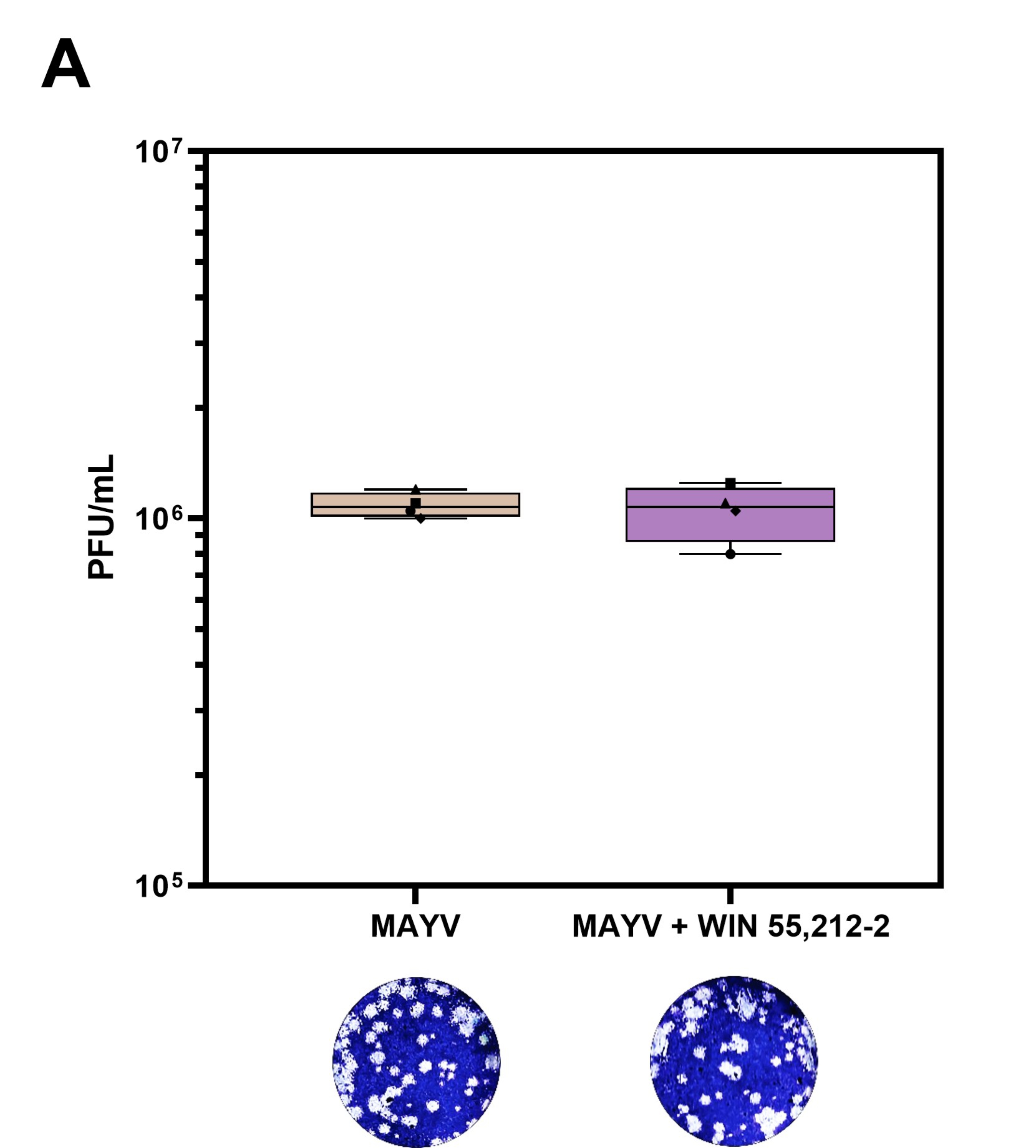

Supplement: Supplementary file 1 [file viruses-18-00662-s001.zip › Figure S2 .jpg]

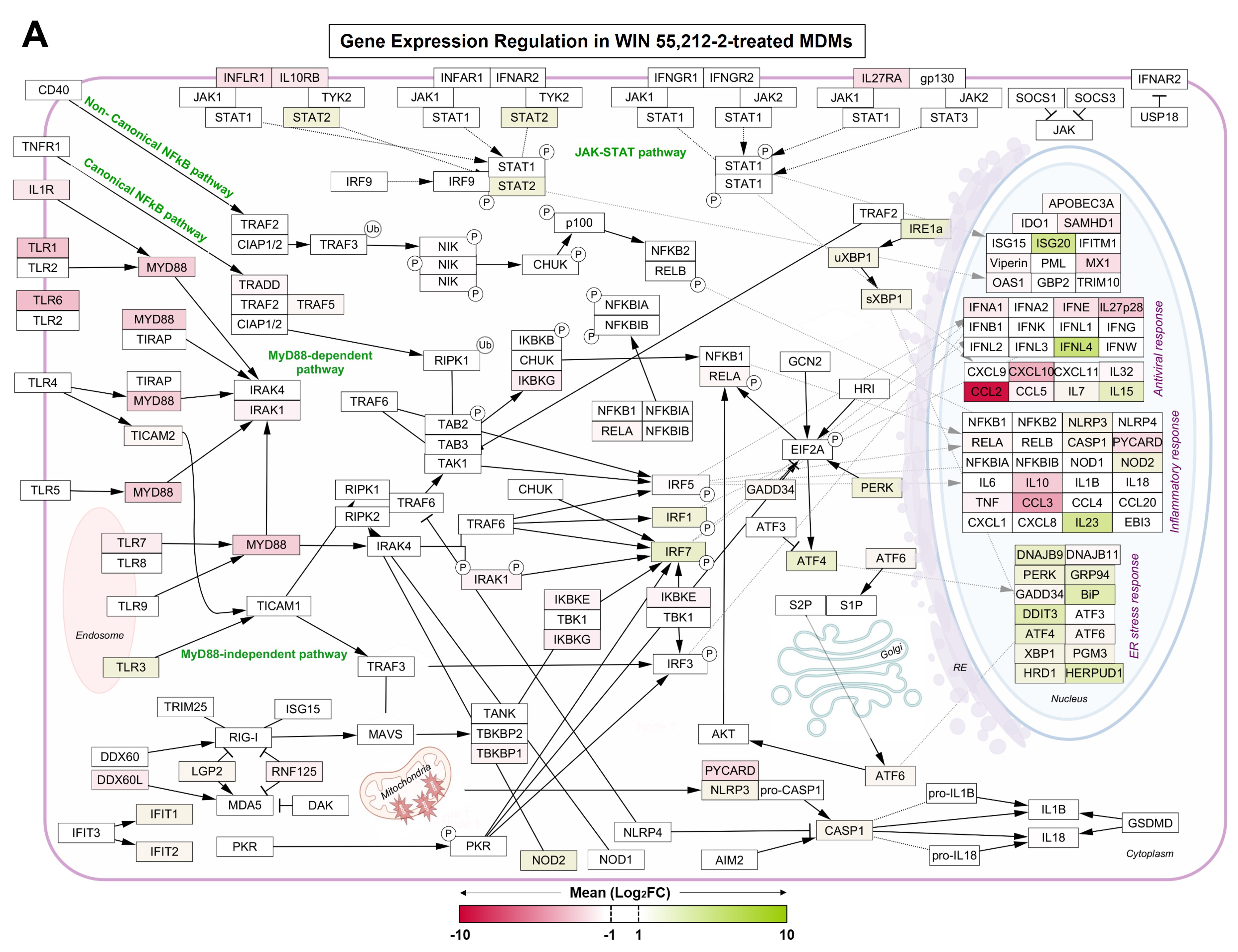

Supplement: Supplementary file 1 [file viruses-18-00662-s001.zip › Figure S3 .jpg]
